# An Unusual Case of Neisseria flavescens/subflava Group Tricuspid Valve Endocarditis in a Patient With Previously Treated Methicillin-Resistant Staphylococcus aureus Endocarditis

**DOI:** 10.7759/cureus.9752

**Published:** 2020-08-14

**Authors:** Anup Solsi, Dawood Findakly, Nawfal Mihyawi, Ayman R Fath

**Affiliations:** 1 Internal Medicine, Creighton University Arizona Health Education Alliance/Valleywise Health Medical Center, Phoenix, USA

**Keywords:** infective endocarditis, neisseria flavescens/subflava, transthoracic echocardiography, mrsa

## Abstract

Infective endocarditis (IE) is classified as an infection of any cardiac valve or endocardial surface and is often associated with high morbidity and mortality. Certain bacteria such as gram-positive cocci and viridans group streptococci have high predilection for both naïve and damaged cardiac valvular tissues, accounting for the majority of IE cases. In very infrequent instances, gram-negative bacteria (GNB), more specifically non-meningococcal, non-gonococcal GNB, have been shown to cause IE. The following is a case of a young male diagnosed with Neisseria flavescens/subflava tricuspid valve endocarditis after being previously treated for Methicillin-resistant Staphylococcus aureus (MRSA) endocarditis.

## Introduction

Infective endocarditis (IE) is an acute or subacute infection of the endocardium of the heart with predilection for heart valve involvement. Overall incidence of IE is approximately 3-10 per 100,000 persons, with recent global mortality rates ranging from 4% to 48% [1,2]. Predisposing risk factors of IE include structural heart disease, presence of prosthetic valves or cardiac devices, such as pacemakers or defibrillators, intravenous (IV) drug use, immunosuppression or recent invasive procedures [3]. Gram-positive cocci, such as Staphylococcus, Streptococcus and Enterococcus species, account for 80%-90% of IE cases, with Staphylococcus aureus and coagulase-negative staphylococci comprising 25% and 22% of cases, respectively [1,4].

Methicillin-resistant Staphylococcus aureus (MRSA), a subtype of S. aureus with greater infectivity due to increased antibiotic resistance, has long been considered one of the leading causes of septicemia/bacteremia and endocarditis. Although common clinical practice is to assess all patients with S. aureus bacteremia for IE, only a fraction ultimately develop IE, with studies showing the prevalence of IE in patients with S. aureus to be 13%-25% [5].

Gram-negative bacteria (GNB) also have been discovered to have a propensity for causing IE, albeit in uncommon situations. The reported incidence of IE caused by GNB varies, with range 1.3%-10% [6]. Of the GNB, the HACEK group of bacteria (Haemophilus parainfluenzae, Aggregatibacter spp., Cardiobacterium spp., Eiknella corrodens, Kingella spp.) are most commonly associated with IE, accounting for 1.2%-3% of cases [7]. In the last few decades, however, the advent of non-HACEK species of GNB causing IE has been identified, with very few cases published in medical literature. In particular, non-meningococcal, non-gonococcal Neisseria species have been recognized as the causal organisms in rare instances.

Here, we present the unique case of a young male with Neisseria flavescens/subflava tricuspid valve (TV) endocarditis who was previously treated for MRSA endocarditis.

## Case presentation

A 27-year-old male with history of IV drug abuse and recent diagnosis of MRSA TV endocarditis at an outside hospital presented from a penitentiary with a two-week history of fevers, cough and diaphoresis. The patient stated he had prolonged hospitalization back in August 2019, in which he was diagnosed with MRSA TV endocarditis and subsequently completed eight weeks of IV antibiotics via peripherally inserted central catheter (PICC) line post-discharge. It was unclear if the vegetation ever resolved. Records showed he was recently prescribed doxycycline; however, the patient denied taking the medication. In jail, he was reportedly diagnosed with pneumonia but refused treatment.

**Figure 1:**
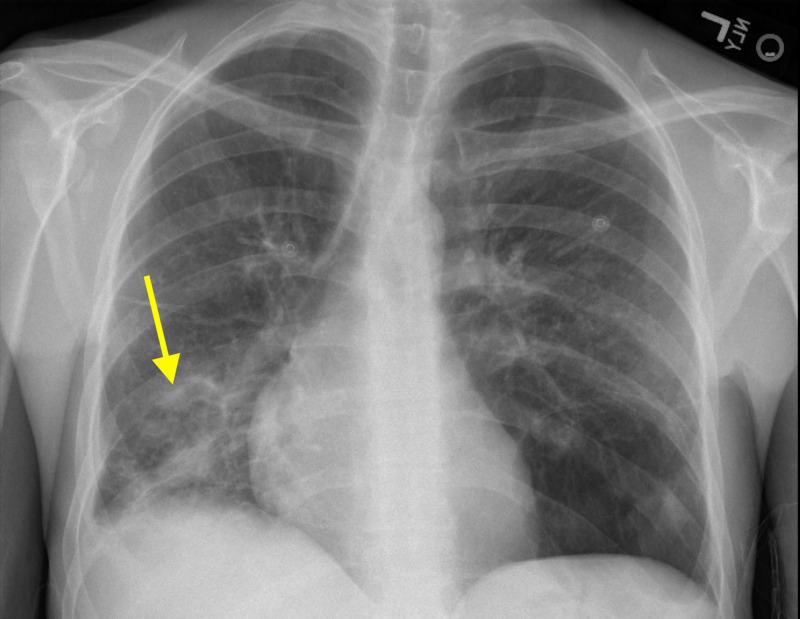
Chest X-ray depicting a predominantly right lower lung infiltrate (yellow arrow) concerning for a developing infectious process.

Further history elicited associated shortness of breath, but no symptoms of chest pain, palpitations, abdominal pain, nausea, vomiting or diarrhea. On admission, the patient was afebrile, mildly tachycardic and hypotensive. Clinical examination revealed a diaphoretic male with a 3/6 holosystolic murmur best heard at the right and left lower sternal borders, a palpable thrill at the left sternal border and a 5 cm laterally displaced apex beat. No peripheral stigmata of IE were found. Breath sounds were coarse in the right lower lobe (RLL), with no egophony. Laboratory investigations were significant for a moderate leukocytosis, iron-deficiency anemia and mild hyponatremia. Urine drug screen was negative. EKG demonstrated a normal sinus rhythm with incomplete right bundle branch block. Chest x-ray (CXR) showed a right lung base infiltrate and questionable left lung bullae (Figure 1).

**Figure 2:**
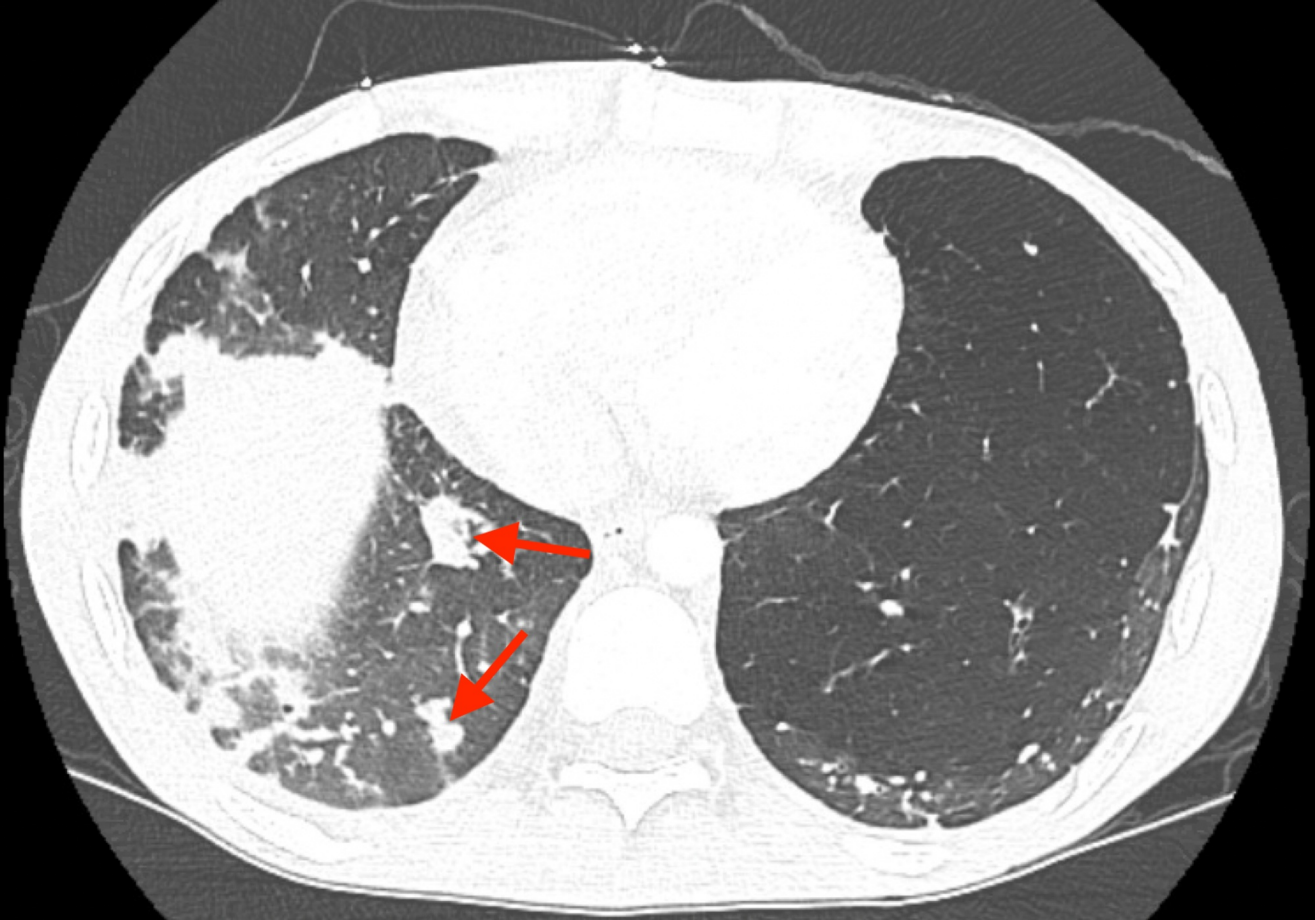
CT scan of the chest showing scattered pulmonary nodules highly suspicious for abscesses secondary to cardiac emboli (red arrows). Multiple other smaller nodules can be seen surrounding the larger nodules.

A CT of the chest was performed for further evaluation, which demonstrated an RLL 2.2-cm cavitary lesion with surrounding consolidation, multiple bilateral solid pulmonary nodules suspicious for abscesses (Figure 2).

**Figure 3:**
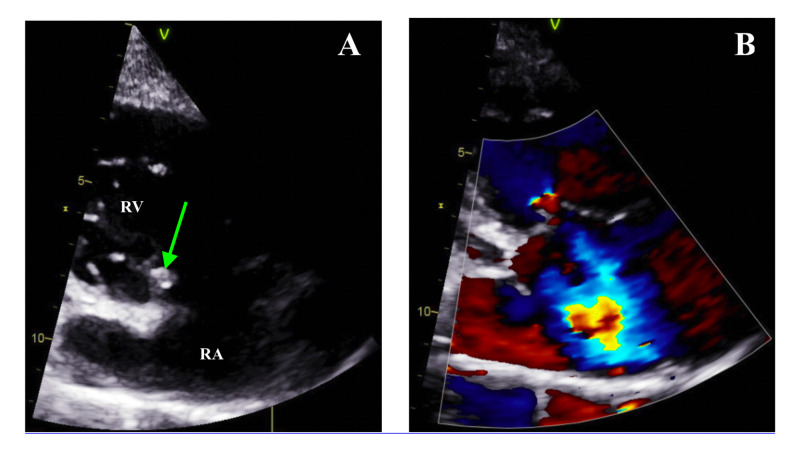
RV inflow view on transthoracic echocardiogram (from parasternal long axis view): (A) large vegetation (green arrow) measuring 21 x 5 mm on the tricuspid valve, (B) severe tricuspid regurgitation, as seen by the blue jet RA: right atrium, RV: right ventricle

The patient was admitted for the management of sepsis, with concern for a community-acquired pneumonia versus recurrent IE with cardioembolic abscesses. After blood cultures were collected, the patient was initiated on vancomycin and cefepime. A transthoracic echocardiogram (TTE) was later obtained and demonstrated severe tricuspid regurgitation with a large TV vegetation measured at 21 x 5 mm (Figure 3). There was diastolic flattening suggestive of right ventricular volume overload but no echocardiographic evidence of right ventricular failure.

Since a vegetation was seen on the TTE, no transesophageal echocardiogram was done. Blood cultures returned positive for N. flavescens/subflava group in both sets. Coccidioidomycosis antibody and QuantiFERON® testing were negative. To further assess the possible etiology of the Neisseria bacteria, a CT maxillofacial was performed, which did not reveal any abscesses. Over the next few days, the clinical condition of the patient improved on antibiotics. Consideration was given for surgical intervention in the form of TV repair, but since there was clinical improvement and no signs of RV failure, no referral for surgery was given. After three days of hospitalization, a PICC line was placed and the patient was discharged back to prison with a six-week course of ceftriaxone.

## Discussion

Non-meningococcal, non-gonococcal species of Neisseria have historically been considered to be non-pathogenic, normal inhabitants of the human nasopharynx/upper respiratory tract [8]. Infectivity of these Neisseria species is very uncommon, but similar to other more virulent strains of GNB, can ultimately lead to severe systemic illness in certain circumstances. Of the relatively few published cases of IE involving non-meningococcal, non-gonococcal Neisseria species, Neisseria elongata and Neisseria sicca have been the most abundant.

N. subflava is characterized as a gram-negative diplococcal saprophyte of the oral cavity that is chromogenic, grows at 22°C, is able to ferment glucose, maltose and sucrose, and produce H_2_S [9]. N. flavescens is very similar to N. subflava, with few microbiological differences that are incompletely understood. From our literature search, only 12 cases of IE caused by N. subflava have been reported, with even fewer numbers for N. flavescens, the first case being reported in 1987 [10].

Suggested risk factors include pre-existing valvular abnormalities or underlying cardiac lesions, immunosuppression (asplenia, chronic hepatitis, HIV, diabetes) and dental conditions (abscesses) [11]. In the past, IV drug use was widely considered to be the most common predisposing risk factor for non-HACEK IE [12]. Recent investigation into non-HACEK organism IE has shed insight onto the possibility of intravascular catheters as a risk factor for IE caused by atypical organisms. In a multicenter study of 26 patients with non-HACEK IE, the most prominent risk factor observed was the presence of an intravascular catheter (e.g. hemodialysis catheter, central venous catheter). Although N. flavescens/subflava and other non-meningococcal, non-gonococcal species of Neisseria are not classified as non-HACEK organisms, there may be a connection between these two groups based on the risk factors predisposing to IE. Our patient was both an IV drug user and had an intravascular catheter in the form of a PICC line, coupled with a previously damaged valve from prior MRSA IE, all likely made him increasingly susceptible to an atypical organism such as N. flavescens/subflava. Besides risk factors, few other similarities between non-meningococcal, non-gonococcal Neisseria species and HACEK bacteria exist. HACEK-IE vegetations have been shown on average to be larger compared to vegetations caused by viridans group streptococci (11.5 mm vs 9 mm), and these vegetations have higher rates of embolization [13]. Our patient was found to have a large vegetation (21 mm) and clinical evidence of embolization, further demonstrating the similarities between HACEK organisms and non-meningococcal, non-gonococcal Neisseria.

Neisseria-induced endocarditis usually results in an acute febrile endocarditis with large vegetations and a destructive process that often causes severe cardiac and systemic complications such as systemic embolization, thrombotic thrombocytopenic purpura, heart failure and myocardial abscesses [14]. The pathogenesis of how this occurs in Neisseria-induced IE remains largely unclear. It was previously thought GNB were rarely implicated in IE due to their inability to form biofilms as well as their relatively low affinity for cardiac endocardial tissue [12]. Newer research has shown N. subflava exhibit biofilm-related phenotypes that are not often seen by laboratory strains [15]. Furthermore, it was shown that similar biofilm phenotypes were observed in Streptococcus mitis, a viridans group Streptococcus also known to cause IE. It is highly possible N. subflava and other Neisseria species are more related to typical IE causing bacteria that was previously thought, but more research is needed to decipher these similarities.

Traditionally, these commensal Neisseria species, including N. flavescens/subflava, were treated with penicillin antibiotics. However, in the 1970s, research studies found that N. flavescens/subflava were capable of beta-lactamase production, thus rendering penicillin antibiotics largely ineffective [10]. Of note, studies of HACEK organisms also have shown their capability to synthesize beta-lactamase, making third-generation cephalosporins the primary antibiotic choice for HACEK-IE treatment [13]. Our patient was treated with cefepime, with subsequent repeat negative blood cultures and discharged on a longer course of ceftriaxone.

## Conclusions

Few reports have described IE caused by non-meningococcal, non-gonococcal GNB. N. flavescens/subflava are commensal, non-pathogenic inhabitants of the human respiratory tract that only become virulent in certain predisposing conditions. Evidence shows some similarities between these bacteria and other gram-positive species known to infect cardiac valves, but further investigation and research is required to understand why then are IE rates so low with these species. 

## References

[1] Cahill TJ, Prendergast BD (2016). Infective endocarditis. Lancet.

[2] Fedeli U, Schievano E, Buonfrate D, Pellizzer G, Spolaore P (2011). Increasing incidence and mortality of infective endocarditis: a population-based study through a record-linkage system. BMC Infect Dis.

[3] Holland TL, Baddour LM, Bayer AS, Hoen B, Miro JM, Fowler VG Jr (2016). Infective endocarditis. Nat Rev Dis Primers.

[4] Cresti A, Chiavarelli M, Scalese M (2017). Epidemiological and mortality trends in infective endocarditis, a 17-year population-based prospective study. Cardiovasc Diagn Ther.

[5] Mylonakis E, Calderwood SB (2001). Infective endocarditis in adults. N Engl J Med.

[6] Reyes MP, Reyes KC (2008). Gram-negative endocarditis. Curr Infect Dis Rep.

[7] Revest M, Egmann G, Cattoir V, Tattevin P (2016). HACEK endocarditis: state-of-the-art. Expert Rev Anti Infect Ther.

[8] Chong Y, Song KS, Lee SY (1975). Neisseria subflava infections: bacteriological aspects of two cases. Yonsei Med J.

[9] Jones DM, Jephcott AE (1990). Neisseria, Branhamella, Moraxella and Kingella. Topley and Wilson's Principles of Bacteriology, Virology and Immunity, 8th Edition.

[10] Sinave CP, Ratzan KR (1987). Infective endocarditis caused by Neisseria flavescens. Am J Med.

[11] Amsel BJ, Moulijn AC (1996). Nonfebrile mitral valve endocarditis due to Neisseria subflava. Chest.

[12] Mercan ME, Arslan F, Alp SO (2019). Non-HACEK Gram-negative bacillus endocarditis. Med Mal Infect.

[13] Ambrosioni J, Martinez-Garcia C, Llopis J (2018). HACEK infective endocarditis: epidemiology, clinical features, and outcome: a case-control study. Int J Infect Dis.

[14] Salvador VB, Chapagain B, Joshi A, Brennessel DJ (2017). Clinical risk factors for infective endocarditis in Staphylococcus aureus bacteremia. Tex Heart Inst J.

[15] Kaplan JB, Fine DH (2002). Biofilm dispersal of Neisseria subflava and other phylogenetically diverse oral bacteria. Appl Environ Microbiol.

